# Combination of JAK2 and HSP90 inhibitors: an effective therapeutic option in drug-resistant chronic myelogenous leukemia

**DOI:** 10.18632/genesandcancer.111

**Published:** 2016-05

**Authors:** Sandip N. Chakraborty, Xiaohong Leng, Bastianella Perazzona, Xiaoping Sun, Yu-Hsi Lin, Ralph B. Arlinghaus

**Affiliations:** ^1^ Department of Translational Molecular Pathology, M.D. Anderson Cancer Center, University of Texas, Houston, TX, USA; ^2^ Department of Laboratory Medicine, M.D. Anderson Cancer Center, University of Texas, Houston, TX, USA

**Keywords:** JAK2, HSP90, Bcr-Abl, CML, drug-resistance, network complex

## Abstract

Recent studies suggest that JAK2 serves as a novel therapeutic target in Bcr-Abl+ chronic myelogenous leukemia (CML). We have reported the existence of an HSP90- associated high molecular weight network complex (HMWNC) that is composed of HSP90 client proteins BCR-ABL, JAK2, and STAT3 in wild type Bcr-Abl+ leukemic cells. Here we showed that the HSP90-HMWNC is present in leukemia cells from CML patients in blast stage, and in Imatinib (IM)-resistant 32Dp210 (T315I) leukemia cells. We found that the HSP90-HMWNC could be disassembled by depleting JAK2 with either Jak2-specific shRNA or treatment with JAK2 inhibitors (TG101209 or Ruxolitinib) and HSP90 inhibitor (AUY922). Combinational treatment with JAK2 and HSP90 inhibitors diminished the activation of BCR-ABL, JAK2 and its downstream targets. As a result, the IM-resistant 32Dp210 T315I cells underwent apoptosis. When administered in mice bearing 32Dp210 T315I leukemia, combinational therapy using Ruxolitinib and AUY922 prolonged the survival significantly. Thus, a combination of JAK2 and HSP90 inhibitors could be a powerful strategy for the treatment of CML, especially in IM-resistant patients.

## INTRODUCTION

Chronic myelogenous leukemia (CML) is a myeloproliferative disorder of blood and bone marrow [1-3]. The Philadelphia chromosome (Ph) is the genetic marker for CML that forms the Bcr-Abl gene due to the reciprocal translocation [t(9;22)(q34;q11)] of chromosome 9 containing the Abl gene and chromosome 22 which contains the Bcr gene [4, 5]. This translocation encodes the BCR-ABL oncoprotein with enhanced tyrosine kinase activity [6, 7]. CML generally progresses through three different phases: chronic phase (CP), accelerated phase (AP), and blast crisis phase (BC). Imatinib mesylate (IM) (Gleevec) is the front line treatment option for early stage CML [8-10]. But unfortunately there are some patients who become resistant to IM treatment mainly due to the mutations in the IM binding site within BCR-ABL, such as T315I, which leads to drug resistance [11-13]. In our previous studies, we found that JAK2 is an important oncogenic target in CML [14-17]. One important function of JAK2 is to stabilize BCR-ABL. The mechanism of BCR-ABL stabilization involves induction of SET by the action of JAK2, which in turn suppresses PP2A. PP2A is critical because it activates SHP1 tyrosine phosphatase. Prevention of SHP1 activity by reduction of PP2A prevents dephosphorylation of tyrosines within BCR-ABL and other proteins. The stability of BCR-ABL appears to be dependent on its being tyrosine phosphorylated [18]. In addition to BCR-ABL, JAK2 also controls other signaling molecules such as LYN by way of the SET, PP2A, and SHP1 pathways [15].

Our findings indicate that IM resistance could be overcome by inhibiting JAK2 activation [14-17]. For example, the gate-keeper mutation T315I form of BCR- ABL is not effectively inhibited by IM and Dasatinib. BCR-ABL T315I continues to activate JAK2 in the presence of IM. We have previously shown that both c-ABL and BCR-ABL activate JAK2 [19]. Moreover, JAK2 plays a critical role in activating several pathways downstream of BCR-ABL including RAS, STAT5, STAT3 and PI-3 kinase/AKT pathways either directly or indirectly by JAK2's ability to stabilize BCR-ABL (as described in ref [14]). Therefore, inhibition of JAK2 by JAK2 inhibitors in IM-resistant cells will prevent JAK2 from activating these pathways even though BCR-ABL is still sending signals to activate JAK2.

Heat shock protein 90 (HSP90) is a molecular chaperone which effects the stability and function of many oncogenic proteins including BCR-ABL [20]. Studies have shown the potential clinical significance of HSP90 inhibitors in treating myeloid disease [21], especially in combination with JAK inhibitors for treating myeloproliferative neoplasms (MPNs) [22] and acute lymphoblastic leukemia (ALL) [23]. NVP-AUY 922 is a novel HSP90 inhibitor that blocks its ATPase activity (hereafter called AUY922). We have shown its effectiveness in inducing cell death in 32Dp210 cells with varying drug-resistant mutations [24]. In this study, we found that HSP90 inhibitor (AUY922) in combination with low doses of JAK2 inhibitors (TG101209 or Ruxolitinib) led to the disassembly of the HSP90- HMWNC in IM-resistant leukemia cells and cell death. Furthermore, leukemic mice bearing IM-resistant CML (T315I) have significantly prolonged their survival when treated with both JAK2 and HSP90 inhibitors (Ruxolitinib and AUY922).

## RESULTS

### The presence of HSP90-containing high molecular weight network complex in blast crisis leukemic cells and in IM-resistant 32Dp210 (T315I) cells

**Figure 1:**
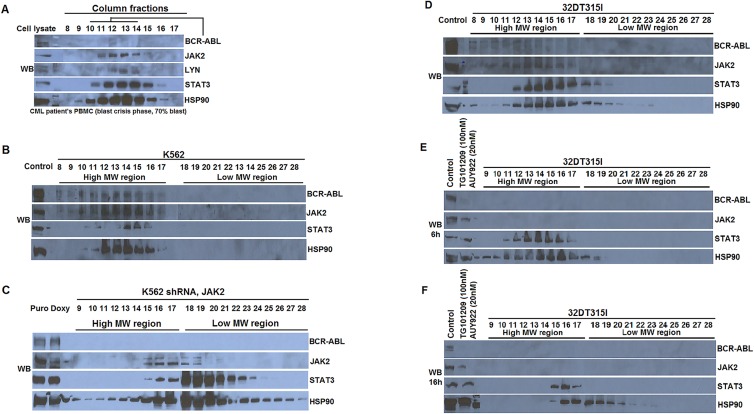
The presence of HSP90-associated High Molecular Weight Network Complex (HMWNC) in CML leukemia cells (A) HSP90-associated HMWNC was detected in the leukemia cells of a CML patient with 70% blast. Cell lysate from the PBMCs in the peripheral blood were fractionated by gel filtration column chromatography. The components of the HMWNC were detected by Western blotting of elutes from fraction number 8 to 17 using antibodies against BCR-ABL, JAK2, LYN, STAT3 and HSP90. (B) The presence of HMWNC in human leukemic cells K562, and its disruption by knocking down Jak2 by Jak2 specific shRNA (C). 3mg of cell lysate from K562 cells or K562 cells with Jak2 knockdown were fractionated by gel filtration column. Elutes from high MW regions (tubes 8 to 17) and lower MW regions (tubes 18 to 27) were analyzed by Western blotting using indicated antibodies. (D) The presence of HMWNC in 32Dp210T315I mouse leukemia cells. (E-F) The breakdown of HMWNC in 32Dp210T315I cells that were treated with JAK2 inhibitor TG101209 (100nM) and HSP90 inhibitor AUY922 (20nM) for either 6hr (E) or 16hr (F).

Our previous studies showed the existence of a high molecular weight network complex (HMWNC) in Bcr- Abl^+^ cell lines 32Dp210 and K562 that contains HSP90, BCR-ABL, JAK2 and STAT3 [20, 24]. To examine the presence of the HMWNC in CML patients, we collected peripheral blood from a CML patient in the blast crisis stage with 70% blasts. Peripheral blood mononuclear cells (PBMCs) were separated using Ficoll. The cell lysate of PBMCs was fractioned using gel filtration column chromatography. We sampled the fractions in the high molecular weight region (6-8 million Dalton in size in fraction numbers 8-17) for the presence of HSP90, BCR- ABL, JAK2, STAT3 and LYN (Figure 1A). We found that the tested proteins co-eluted in the high molecular weight fractions, a pattern similar to our previous observation in K562 and 32Dp210 cells [20], indicating the existence HSP90-HMWNC in leukemic blast cells from CML patients.

Unlike K562 (a blast crisis CML cell line), we observed that the level of BCR-ABL protein was low in the fractions 11, 12, and 13 of the patient's sample (Figure 1A). This lower level of Bcr-Abl might be due to the low level of Bcr-Abl transcripts in leukemia cells and the high protease activity in the granulocytes in the patient's sample. In addition, we detected the presence of HSP90- HMWNC that contained BCR-ABL, JAK2, and STAT3 in the high molecular weight region in the cell lysate from Imatinib (IM)-resistant 32Dp210 T315I mouse leukemia cells (Figure 1D).

### Destruction of the HSP90-HMWNC by depleting JAK2 with Jak2-specific shRNA or combinational treatment with JAK2 and HSP90 inhibitors

Our previous studies showed that the HSP90- HMWNC is sensitive to inhibitors of BCR-ABL, JAK2 or HSP90 in wild type Bcr-Abl^+^ cells [20]. To address the issues of non-specificity of using chemical inhibitors [25], we analyzed the HSP90-HMWNC in K562 cells with JAK2 knocked down using Jak2-specific shRNA [26]. Unlike the parental cells (Figure 1B), reducing the expression of JAK2 by shRNA in K562 cells shifted the signaling molecules BCR-ABL, JAK2 and STAT3 in HSP90-HMWNC from the high molecular weight region to the lower molecular weight region, suggesting the disassembly of the HSP90-HMWNC (Figure 1C).

**Figure 2:**
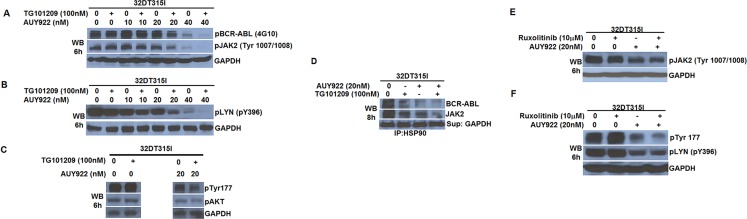
Combinational treatment using both HSP90 and JAK2 inhibitors enhanced the inhibition of the BCR- ABL signaling in IM-resistant 32Dp210 T315I cells (A-D) IM-resistant 32Dp210 T315I cells were treated with JAK2 inhibitor TG101209 (100nM) with different dosages of HSP90 inhibitor AUY922 (0-40nM) for 6hr. Western blotting was performed using antibodies against pBCR-ABL (4G10) and pJAK2 (Tyr1007/1008) (A); pLYN (B); BCR-ABL pTyr177 (C); and pAKT (D). (E) Decreased levels of BCR-ABL and JAK2 in HSP90-immunoprecipitation complex from 32Dp210 T315I cells treated with JAK2 inhibitor or HSP90 inhibitor, or both; (E-F) A different JAK inhibitor Ruxolitinib (10μM) showed synergistic effect with HSP90 inhibitor AUY922 (20nM) in blocking JAK2 activity as measured by pJAK2 (Tyr1007/1008) (E), BCR-ABL activity as measured by pTyr177, and LYN activity as measured by pTyr396 (F).

We have showed that the HSP90-HMWNC is sensitive to HSP90 inhibitor AYU922, which leads to the disassembly of the multiprotein complex and cell death in 32Dp210 cells [24]. When treating IM-resistance 32Dp210 T315I cells with either JAK2 inhibitor TG101209 (100nM) or HSP90 inhibitor AUY922 (20nM) alone, the association of JAK2 with HSP90 was decreased (Figure 2D). The loss of JAK2 and BCR-ABL in HSP90 immunoprecipitation (IP) complex was more apparent in cells treated with both drugs (Figure 1D, lane 4). Furthermore, this combinational treatment led to the disassembly of the HSP90-HMWNC, as evidenced by the analysis using gel filtration chromatography (Figure 1F). In cells treated with both drugs for a shorter time (6hr), HSP90-HMWNC was relatively intact (Figure 1E). When the cells were treated for longer hours (16hr), the components of the HMWNC shifted towards a lower molecular weight region (Figure 1F). Interestingly, HSP90 was found to associate with some members of the complex such as STAT3 which was co-eluted in the same fractions (Figure 1F).

### Combinational treatment of HSP90 and JAK2 inhibitors prevents the Bcr-Abl signaling in IM- resistant 32Dp210 T315I cells

In our previous publications we have shown that JAK2 regulates key signaling molecules in Bcr-Abl-driven pathways including LYN, AKT, and pTyr177 of BCR-ABL [14-16, 20], suggesting that JAK2 is a potential therapeutic target in CML [14, 15, 17, 27]. However, the relatively high concentration that is needed for the effectiveness of JAK2 inhibitors limits its clinical application due to the potential side effects. Knowing that the HSP90 inhibitor AUY922 disrupts the HSP90-HMWNC, we wondered whether an additive inhibition effect could be achieved when both JAK2 and HSP90 inhibitors are used simultaneously at a relatively low concentration. We tested this hypothesis in IM-resistant 32Dp210 T315I cells. We applied a fixed dose of TG101209 (100nM) and titrated the concentration of AUY922 from 0 to 40nM in 32Dp210 T315I cells, followed by measuring the activation status of pBCR-ABL (Y177), pJAK2(Y1007/1008), pLYN(Y369) and pAKT using phospho-specific antibodies (Figure 2A-C). When using the JAK2 inhibitor TG101209 at 100nM alone, the effect on the activities of pBcr-Abl, pJAK2, pLYN and pAkt was minimal. When treating with HSP90 inhibitor AUY922 alone, the activities of these signaling molecules started to decrease at 20nM condition and further diminished at 40nM treatment. Importantly, we observed the additive effect of TG101209 (100nM) together with either 20nM or 40nM of AUY922, in which the activation of Bcr-Abl, JAK2, LYN or AKT was greatly inhibited. This result suggested that the combination of HSP90 and JAK2 inhibitors in a relatively low dosage of each inhibitor could be more effective in blocking a number of BCR-ABL signaling molecules.

This combinational inhibition was also observed using a different JAK1/2 inhibitor Ruxolitinib (Figure 2E and 2F). However, a much higher concentration of Ruxolitinib (10μM) in combination of AUY922 (20nM) was needed for the effective inhibition of pJAK2, pBCR- ABL and pLYN.

### Inducing apoptosis in IM-resistant T315I cells treated with both JAK2 and HSP90 inhibitors

**Figure 3:**
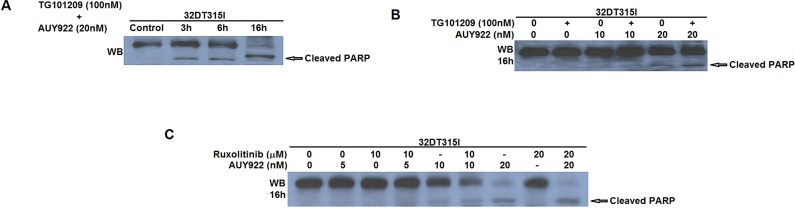
Combined treatment of JAK2 and HSP90 inhibitors in Bcr-Abl+ IM-resistant cells induced apoptosis (A) 32Dp210 T315I cells were treated with TG101209 (100nM) and AUY922 (20nM) for 0-16hr. Cell lysate were probed with anti-PARP antibodies to indicate the apoptosis levels as measured by the presence of cleaved PARP; (B) Induction of apoptosis was enhanced in 32Dp210 T315I cells when treated with both TG101209 and AUY922 for 16hr; (C) A different JAK inhibitor Ruxolitinib (0-20μM) and showed similar synergistic effect with AUY922 (0-20nM) in inducing apoptosis in 32Dp210 T315I cells.

Surprisingly, we observed that the proliferating rate of K562 cells with reduced JAK2 expression by Jak2-specific shRNA was not apparently different from the parental cells, which may be due to the incomplete depletion of JAK2 (data not shown). However, when 32Dp210 T315I cells were treated with both TG101209 (100nM) and AUY922 (10-20nM) for 3-16hrs, the amount of apoptotic cells were gradually increased as measured by the level of cleaved PARP (Figure 3A). Furthermore, either inhibitors alone was less capable to induce apoptosis in 32Dp210 T315I cells, compared to combinational treatment with both TG101209 and AUY922 (Figure 3B), suggesting the synergistic effect of using both JAK2 and HSP90 inhibitors at a relatively low concentration to kill IM-resistant leukemia cells. This additive effect in inducing apoptosis in 32Dp210 T315I cells was also observed when using a different JAK1/2 inhibitor Ruxolitinib with TG101209, albeit at a higher amount of Ruxolitinib (10 or 20μM) (Figure 3C). This result may have a clinical implication to reduce the side effects of using the JAK inhibitor at a higher dose.

### Combinational therapy of Ruxolitinib and AUY922 significantly prolonged the survival in mice bearing 32Dp210 T315I leukemia

**Figure 4:**
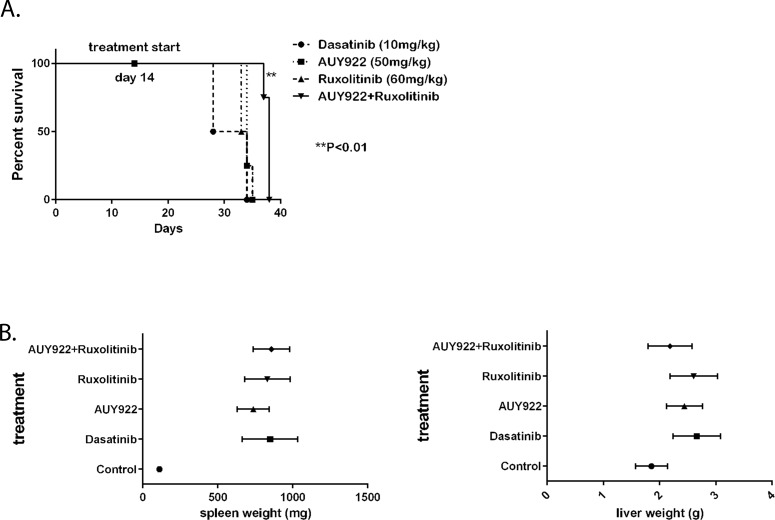
The survival of mice bearing 32Dp210 T315I leukemia was significantly improved when treated with combination of HSP90 and JAK2 inhibitors (A) Survival curve of the mice experiment (**P<0.01). Drug treatment started at fourteen days after injecting 32Dp210 T315I cells into C3HJ mice via tail vein. (B) Comparison of the weight of spleens (left) and livers (right) from the four groups of mice.

To test the effectiveness of combination of HSP90 and JAK2 inhibitors *in vivo*, we employed the syngeneic mouse model using C3H mice and luciferase-labeled 32Dp210 T315I cells. After 14days of injecting 2 million leukemic cells via tail vein, mice developed a CML- like disease with leukemic cells spreading to bone, liver and spleen detected using live animal imaging. We then divided the animals into four groups for treatment with Dasatinib (10mg/kg), or AUY922 (50mg/kg), or Ruxolitinib (60mg/kg), or the combination of AUY922 and Ruxolitinib. The drugs were delivered by oral gavage three days a week. As expected, Dasatinib could not block the growth of p210 T315I leukemic cells, and the mice treated with Dasatinib died between 28 to 32 days after initial cell injection. However, combinational treatment with AUY922 and Ruxolitinib significantly prolonged the survival for an average of 7 days (**P<0.01) (Figure 4A). Treatment with AUY922 or Ruxolitinib alone also prolonged the survival but to a lesser extent (Figure 4A). Mice in all treatment groups eventually succumbed to extensive leukemic cells infiltrated into the spleens and livers (Figure 4B).

## DISCUSSION

These studies demonstrated that a combination of a JAK2 inhibitor and a HSP90 inhibitor induced apoptosis in IM resistant T315I Bcr-Abl leukemic cells. The advantage of this combination treatment is that it requires lower doses of JAK2 inhibitor to achieve significance in survival by avoiding the toxicity of using high amount of JAK2 inhibitor.

Our previous studies showed that in addition to Bcr-Abl, the JAK2 tryrosine kinase is a key signaling molecule in Bcr-Abl+ CML cells [14, 15, 17, 27, 28]. Mutations in JAK2 have been identified in patients with varies forms of myeloproliferative disorders. The resultant constitutive activation of JAK-STAT pathway is critical for the cytokine-independent growth of leukemia cells. In the clinic, FDA-approved JAK1/2 inhibitor Ruxolitinib effectively improved the blood count and splenomegaly in myeloproliferative neoplasia (MPN) patients. However, it did little to decrease the burden of leukemia cells, and high dose of Ruxolitinib generates toxic effects on normal hematopoiesis [29, 30]. We found that JAK2 inhibition in 32Dp210 cells leads to an increase in JAK2 protein, making it difficult to inhibit JAK2 with low doses of JAK inhibitors (unpublished findings). Thus, higher concentrations of JAK2 inhibitors are needed to interfere effectively with JAK2 activity (Lin, Chakraborty and Arlinghaus, in preparation). This high level of JAK2 inhibitors undoubtedly leads to inhibition of off-target proteins [25].

Drug resistance has been observed in patients after chronic Ruxolitinib treatment. To overcome its limited efficacy, the Levine group combined HSP90 inhibitor PU-H71 with Ruxolitinib to treat mice that developed a lethal myeloproliferative neoplasia induced by expression mutant MLPW515L [22]. Kucine et al. also showed PU- H71 prolonged the survival in mice with Roxulitinib-resistant JAK-mutant acute lymphoblastic leukemia (ALL) [23]. In our study, we tested the combinational treatment of Ruxolitinib with a different HSP90 inhibitor AUY922 in mice bearing IM-resistant Bcr-Abl+ 32Dp210 T315I cells. We found this combinational drug approach significantly prolonged the survival of mice bearing IM- resistant leukemia.

Moreover, we provided a molecular mechanism underlying the effectiveness of using HSP90 inhibitor in treating drug-resistant Bcr-Abl+ leukemia. We detected a high molecular weight network complex (HMWTNC) (6-8 million Dalton) in leukemia cells. HSP90 inhibition by AUY922 combined with the JAK2 inhibitor disrupts this HSP90-HMWNC and leads to cell death. The collapse of HSP90-HMWNC might be the initial step that leads to the degradation of HSP90 and JAK2 protein as well as other members of the network complex. Importantly, we also detected HMWNC in fresh uncultured Bcr-Abl+ blast crisis CML patient cells. In this case, all the typical components of the HMWNC were detected, but there were relatively low levels of Bcr-Abl. This low level of Bcr-Abl is likely due to the less copy numbers of Bcr-Abl transcripts in patient samples compared to that in 32Dp210 cells, and high protease activity of granulocytes [31, 32].

In addition to their co-existence in HMWNC, JAK2 may regulate HSP90 at transcription level. Ammirante *et al* reported the involvement of NF-κB in HSP90 regulation, and Prinsloo *et al* stated HSP90 regulation by STAT3 [33, 34]. In previous reports, we and others reported regulation of both NF-κB and STAT3 by JAK2 [15, 16, 35]. Furthermore, Schoof *et al* showed that the activities of JAK2 and STAT3 are regulated by HSP90 [36]. In our study, we showed that JAK2 co-immunoprecipitated with HSP90, and this interaction was mostly inhibited by the presence of both JAK2 and HSP90 inhibitors (Figure 2D). Future investigation of the impact at the transcriptional and activities of JAK2 and HSP90 will lead us to better understand the mechanism of enhancement by low doses of combined inhibitor treatment.

Bcr-Abl T315I + cells have a gate keeper mutation that resists to treatment of Gleevec (Imatinib mesylate) and Dasatinib. In our animal studies, JAK1/2 inhibitor Ruxolitinib alone improved the survival in mice bearing T315I leukemia compared to the group treated with Dasatinib but not to a significant extent. However, when combining the JAK2 inhibitor with HSP90 inhibitor AUY922, our results showed a synergistic effect of the two inhibitors which significantly prolonged the survival with no toxic effects observed. Taken together, our data provided an effective therapeutic option for treating drug-resistant CML patients. The underlying molecular mechanism involves HSP90-HMWNC containing the JAK2 kinase. Clinical trials using both JAK2 and HSP90 inhibitors may provide a possible targeted therapy for IM- resistant CML patients.

These new findings in mice raise some important questions, which we have not yet answered. We have not yet determined whether JAK2 and HSP90 signaling were inhibited in leukemic cells from the treated mice. The amount of leukemia cells from the treated mice was not yet sufficient for the measurements of the status of the HSP90-HMWNC, as we showed in the in vitro experiment. Nevertheless, the mice results provided the proof of principle of using both JAK2 and HSP90 inhibitors to treat the IM-resistant BCR-ABL positive leukemia.

## MATERIALS AND METHODS

### Reagents and antibodies

AUY922 compound (NVP-AUY922) was kindly provided by the Novartis Institution for BioMedical Research, and was later purchased from the LC laboratories (N-5300). Imatinib, Dasatinib (D3307) and Ruxolitinib phosphate (R6688) were purchased from the LC laboratories. For in vitro studies, AUY922, Imatinib, Dasatinib, Ruxolitinib and TG101209 were dissolved in DMSO (dimethyl sulfoxide) and diluted with sterile PBS to yield a stock concentration of 10mM. For in vivo studies, Dasatinib and AUY922 were prepared in 80mM Citric Acid. Ruxolitinib phosphate was dissolved in PBS+0.1% Tween-20. The antibodies used for this study were against: c-ABL (Cell Signaling, Cat # 2862), phospho-JAK2(Y1007) (Millipore, Cat # 04-1098), JAK2 (Santa Cruz, Cat# sc-294), STAT3 (Cell signaling, Cat# 9132), AKT (Santa Cruz, Cat# sc-1619), HSP90 (Santa Cruz, Cat# sc-7947), phosphotyrosine (4G10) (Millipore, Cat# 05-321), pSRC family (Tyr 416) (Cell Signaling, Cat# 2113S), pBCR (Y177) (Cell Signaling, Cat# 3901S).

### Cell culture

The human leukemic cell line K562 and mouse leukemic cell lines 32Dp210 and 32Dp210 T315I were cultured in RPMI 1640 medium supplemented with 10% fetal bovine serum (FBS), 100mg/L penicillin/ streptomycin, and 2mM Glutamine. Cells were cultured in a humidified incubator at 37°C with 5% CO_2_.

### Gel filtration column chromatography, Immunoprecipitation and Western blotting

Gel filtration column chromatography was performed as described [20, 24]. The column was 50cm in length × 0.7cm in diameter (Econo column, Bio-Rad, Cat # 737-0752) packed with Superose 6 prep grade gel filtration column material (Amersham-Biosciences, GE Healthcare, Cat # 17-0489-01). The elution buffer was 30mM HEPES (pH 7.4) containing 150mM NaCl, 10% glycerol and 0.5% NP-40. Cell lysates were prepared following the previous protocol [28]. Elutes from the gel filtration column were collected (500μl) using fraction collector at 4.56 ml/h at 4°C. Immunoprecipitation and Western blotting of the fractions were performed as described [28].

### In vivo mouse experiments

100ul of 2×10^6^ luciferase-labeled 32Dp210 T315I cells were injected via tail vein of 6wk-old C3H mice purchased from the Jackson Laboratory. The development of leukemia was monitored using live animal imaging using (Xenogen IVIS 100). Two weeks after injection, mice were randomly divided into four groups and were given Dasatinib (10mg/kg), AUY922 (50mg/kg), Ruxolitinib (60mg/kg), or the combination of AYU922 (50mg/kg) and Ruxolitinib (60mg/kg) by oral gavage in volumes of 100-180ul on Monday, Wednesday and Friday. The progression of leukemia in these mice was monitored by live animal imaging twice a week. The moribund mice were euthanized. Spleen and liver tissues were collected for pathology analysis. The survival curves were generated using GraphPad Prism6 software.
